# First-in-human dosimetry and biodistribution of [^89^Zr]Zr-oxine-labeled autologous leukocytes using PET/CT

**DOI:** 10.1007/s00259-026-08158-w

**Published:** 2026-10-02

**Authors:** Kadir Gultekin, Jennifer L. Bartels, Carlos E. Cardenas, Yogesh Kumar, Grayson R. Gimblet, Chloe L. Jones, Christina Bankston, Charlotte D. Jeffers, Sharon L. White, Suzanne E. Lapi, Jarred W. Younger, Jonathan E. McConathy

**Affiliations:** 1 Department of Biomedical Engineering, University of Alabama at Birmingham, Birmingham, AL 35294 USA; 2 Department of Radiology, University of Alabama at Birmingham, Birmingham, AL 35294 USA; 3 Department of Radiation Oncology, University of Alabama at Birmingham, Birmingham, AL USA; 4 Department of Psychology, University of Alabama at Birmingham, Birmingham, AL USA; 5 Department of Psychiatry and Behavioral Neurobiology, University of Alabama at Birmingham, Birmingham, AL USA

**Keywords:** PET, Cell tracking, Dosimetry, Leukocytes, Zirconium-89, Oxine

## Abstract

**Purpose:**

Cell tracking with zirconium-89 PET has the potential for widespread clinical and research applications, including imaging infection and inflammation and tracking cell-based therapies. This prospective first-in-human clinical trial assessed the dosimetry and safety profile of [^89^Zr]Zr-oxine-labeled autologous leukocytes in healthy female participants.

**Methods:**

Four healthy adult female participants (ages 37–53) received 10.9 ± 2.9 MBq of [^89^Zr]Zr-oxine-labeled autologous leukocytes and underwent whole-body PET/CT imaging at five time points (0, 4, 24, 48, and 72 h). Regions of interest were automatically segmented on CT using a machine learning-based approach and manually refined. Time-integrated activity coefficients were calculated using Simpson’s rule, with physical decay assumed beyond the final time point. Mean absorbed-dose and effective-dose coefficients were estimated using OLINDA 2.1.

**Results:**

Administration was well-tolerated among all participants. The highest mean absorbed-dose coefficients (mGy/MBq) with standard deviation were observed in the spleen (3.52 ± 1.38), liver (2.1 ± 0.45), adrenals (1.62 ± 0.24), uterus (1.48 ± 0.98), and kidneys (1.36 ± 0.12). The mean effective-dose coefficient was 0.92 ± 0.06 mSv/MBq, numerically lower than the predicted value of 1.14 mSv/MBq based on the distribution of [^111^In]In-oxine-labeled leukocytes in adult females (*P* = 0.006, one-sample t-test). The use of [^89^Zr]Zr-oxine-labeled leukocytes enabled the visualization of leukocyte distribution and temporal dynamics.

**Conclusion:**

This study demonstrates the safety and favorable dosimetry of [^89^Zr]Zr-oxine-labeled leukocytes in healthy humans. These results support the further use of this approach for imaging autologous leukocytes using PET in a range of clinical and research settings.

**Trial registration:**

ClinicalTrials.gov NCT03807973. Registered January 16, 2019.

**Graphical Abstract:**

**Figure:**
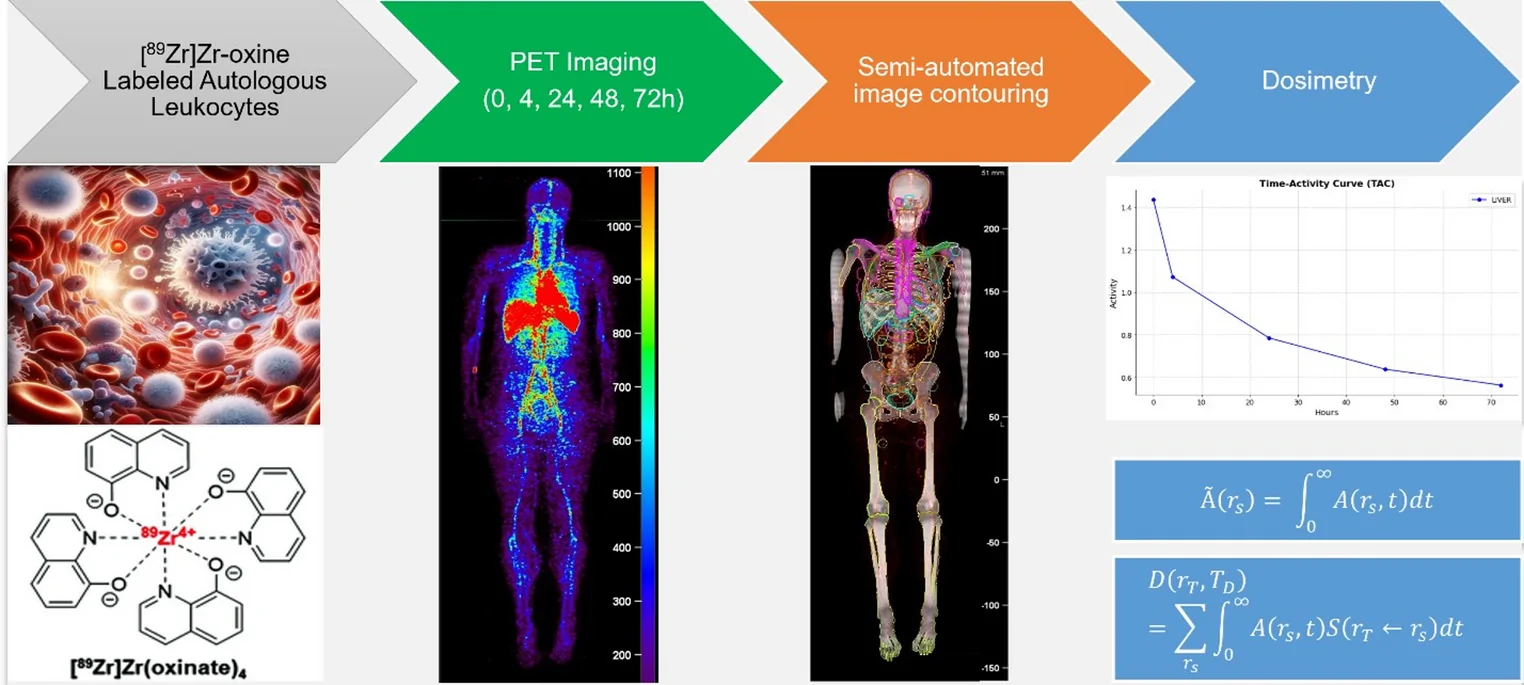

**Supplementary Information:**

The online version contains supplementary material available at https://doi.org/10.1007/s00259-026-08158-w.

## Introduction

Radiolabeling of autologous leukocytes with the gamma-emitting radioisotopes technetium-99m (^99m^Tc, t_1/2_ = 6 h) and indium-111 (^111^In, t_1/2_ = 2.8 d) was first reported in the mid-1970s [1]. Following the first-in-human imaging with [^111^In]In-oxine-labeled leukocytes [2], single-photon emission computed tomography (SPECT) imaging of autologous leukocytes found widespread clinical application imaging infection [3–5] and other inflammatory processes [6, 7]. The recent development of cell-based therapies, such as chimeric antigen receptor (CAR) T-cells, has increased interest in labeling cell populations for real-time therapy response assessment [8–10].

While SPECT radiopharmaceuticals such as, [^111^In]In-oxine and [^99m^Tc]Tc-hexamethylpropylenamine-oxime (^99m^Tc-HMPAO) have traditionally been used for leukocyte imaging, the higher sensitivity, better spatial and temporal resolution of PET imaging compared to SPECT [11–14], has motivated the development of a PET imaging approach for the radiolabeling of autologous leukocytes [15–18]. This approach has been made possible in recent years by the routine production of long-lived PET radioisotopes, such as zirconium-89 (^89^Zr, t_1/2_ = 3.3 d) [19, 20]. With a comparable physical half-life to ^111^In, [^89^Zr]Zr-oxine is well-suited for tracking autologous leukocytes over several days to a week after administration [21, 22]. Preclinical studies with [^89^Zr]Zr-oxine radiolabeled autologous leukocytes in murine [23–25], non-human primate models [26, 27] and initial human studies [28] have demonstrated the feasibility of this approach. A recent first-in-human infection imaging study using ^89^Zr-labeled leukocytes with a cell-surface chelator approach (Df-Bz-NCS) further supports the translational potential of PET leukocyte tracking in patients with confirmed infection [28]. However, intracellular labeling with [^89^Zr]Zr-oxine differs fundamentally from cell-surface chelator strategies in terms of radiochemistry, cellular retention, and potential in vivo redistribution. As a result, these approaches are not necessarily equivalent in terms of dosimetry and dedicated human dosimetry data for intracellular [^89^Zr]Zr-oxine-labeled leukocytes remain lacking. There is also great interest in using PET to track cell-based therapies such as CAR T-cells in cancer patients, and the labeling approach used in this dosimetry study could have applications beyond autologous leukocytes.

This study presents the first-in-human radiation dosimetry and safety evaluation of [^89^Zr]Zr-oxine-labeled autologous leukocytes in a cohort of four healthy adult female participants. This dosimetry and feasibility data are critical for applying this cell imaging technique in larger human studies. These findings also support the use of this approach to image immune cells throughout the body, including within the central nervous system (CNS) in conditions associated with neuroinflammation leading to increased leukocyte migration into the CNS or in the setting of blood-brain barrier (BBB) disruption [29]. The focus on female subjects in this dosimetry cohort was motivated by two primary considerations. Firstly, the predicted dosimetry of [^89^Zr]Zr-oxine leukocytes in humans extrapolated from data on [^111^In]In-oxine leukocytes suggests a higher effective-dose coefficient in females (1.14 mSv/MBq) compared to that in males (0.855 mSv/MBq) [30]. Secondly, the prevalence of Myalgic encephalomyelitis/chronic fatigue syndrome (ME/CFS), which is a key target for the initial planned application of [^89^Zr]Zr-oxine-labeled leukocytes, is higher in females [31]. These factors underscore the rationale for selecting a female cohort in this study.

## Materials and methods

### Patient population and study design

This first-in-human, prospective, single-center phase 1 study (NCT03807973) aimed to determine the radiation dosimetry of [^89^Zr]Zr-oxine-labeled leukocytes in four healthy adult female volunteers. The study protocol, informed consent, advertisements, and imaging procedures were approved by the institutional review board (IRB) at the University of Alabama at Birmingham (UAB), Birmingham, AL, USA, on May 5, 2021, and the study conducted under an Investigational New Drug protocol (IND136462).

**Fig. 1:**
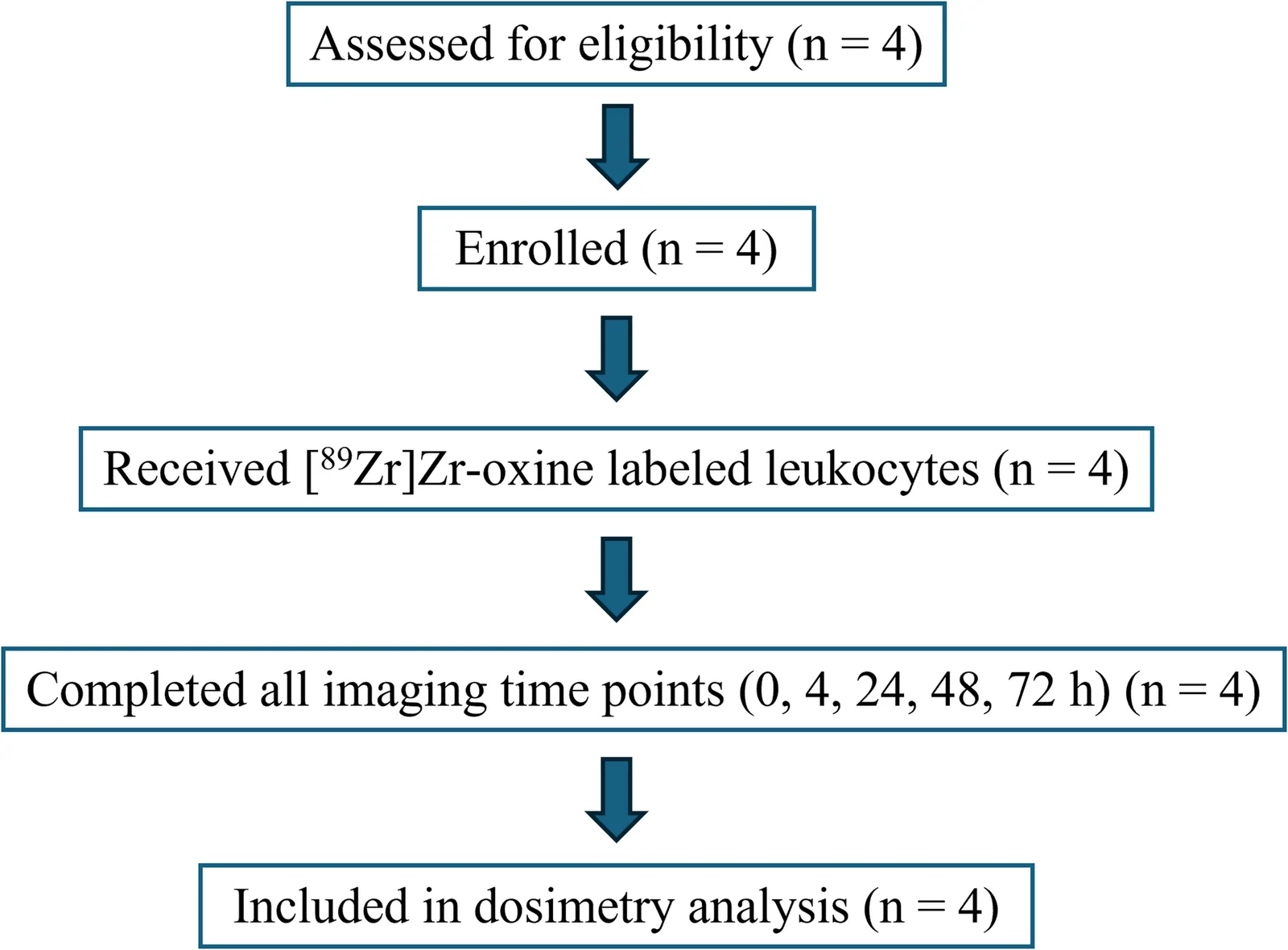
Study flow diagram. Flow of participants through eligibility assessment, enrollment, PET/CT imaging, and dosimetry analysis in this prospective first-in-human study of [^89^Zr]Zr-oxine-labeled autologous leukocytes

Participant flow through the study is shown in Fig. 1. Participation criteria included healthy female adults between 18 and 65 years of age. Major exclusion criteria included pregnancy, lactation, and contraindications to PET/CT imaging. A full list of inclusion and exclusion criteria are provided in Table 1.

**Table 1:** Inclusion and exclusion criteria

Inclusion criteria	Exclusion criteria
• Age 18–65 years • Healthy volunteers	• Pregnancy • Lactation • Individuals unable to participate in the imaging portion due to severity of their condition • Chronic infectious disease (e.g. HIV, HCV) • Viral or bacterial illness requiring medical attention and/or antibiotics within one month of the study • Diagnosis of cancer, including leukemia • Blood or blood clotting disorder • Diagnosis of autoimmune disease • Positive urine β-hCG test the day of procedure or serum β-hCG test within 48 h prior to [^89^Zr]Zr-oxine-labeled leukocyte administration • Currently enrolled in a clinical trial utilizing experimental techniques • Any pathological state or medication regimen that substantially alters biodistribution

### Preparation of [^89^Zr]Zr-oxine

[^89^Zr]Zr-oxine was prepared based on a method developed by our group in the UAB Cyclotron PET Production Facility under United States Pharmacopeia (USP) Chapter < 823 > guidelines [15]. Briefly, [^89^Zr]Zr-oxalate was neutralized and mixed with oxine in dichloromethane followed by vortexing for 30 min at room temperature. The organic layer containing [^89^Zr]Zr-oxine was separated, dried and formulated with DMSO, polysorbate 80, HEPES, and saline. The sterile-filtered final product (> 37 MBq/mL) met quality control specifications, including radiochemical purity ≥ 95%, radionuclidic purity ≥ 99.5%, dichloromethane < 600ppm, dimethyl sulfoxide 15–50 mg, bacterial endotoxin ≤ 175 EU/vial, pH range of 7-7.5, and sterility [15].

### Labeling and study protocol

Autologous leukocytes were radiolabeled using an ex vivo procedure performed at a licensed commercial radiopharmacy under USP Chapter < 797>. Approximately 120 mL of peripheral blood was collected from each participant and transported for processing. Red blood cells were allowed to sediment, and leukocyte-rich plasma was centrifuged to isolate a leukocyte pellet. Isolated leukocytes were incubated with [^89^Zr]Zr-oxine (37–51.8 MBq), washed to remove unbound activity, and resuspended to a final injectable volume of 3–4 mL, corresponding to an administered activity of 7.4–18.5 MBq. Labeled leukocytes were returned to the study site and reinjected intravenously within 5 h of blood collection or within 3 h of radiolabeling, whichever occurred first.

Each labeled leukocyte preparation underwent quality-control testing before clinical administration according to the IND product release specifications. Quality-control testing included assessment of labeling efficiency, radiochemical purity, leukocyte viability before and after labeling, in vitro ^89^Zr efflux at the 3-hour release time point, sterility, and bacterial endotoxin testing. Only products meeting all predefined release criteria were administered to study participants. A summary of the radiolabeling quality-control results is provided in Supplementary Table S1 [15].

The study consisted of five visits. At the initial visit, eligibility was confirmed through written informed consent, and baseline vital signs were recorded and reassessed at each subsequent visit. Women of childbearing potential underwent pregnancy testing prior to radiotracer administration. Adverse events were assessed at each visit. Participants underwent whole-body PET/CT imaging immediately after administration and again at 4–6 h on the same day, with additional imaging performed at 24, 48, and 72 h, for a total of five imaging time points. At each imaging time point, venous blood samples (2–5 mL) were collected, and whole blood and plasma activity were measured using a gamma counter (PerkinElmer Wizard 2480).

### Imaging protocol

Whole-body PET/CT scans were performed on a GE Discovery 710 PET/CT system, without patient fasting or special preparation. Images were acquired from the skull vertex to the toes with 4–6 min per bed position at five time points (immediate, 4–6 h, 24 h, 48 h, and 72 h post-injection).

PET images were reconstructed using ordered-subsets expectation maximization (OSEM) with standard corrections for radioactive decay, random coincidences, and scatter. Vendor-specific parameters included VUE Point High Definition (VPHD) reconstruction, SharpIR, 24 subsets, 2 iterations, a filter cutoff of 6.4 mm and a 192 × 192 image matrix size with 3.27 mm slice thickness for PET slices.

For the initial scans, a standard CT protocol was used to balance image quality and radiation dose, using a helical scan with 0.5-second rotations, a tube voltage of 140 kVp, and tube current modulation capped at 200 mA [32, 33]. Subsequent CT scans employed a low-dose protocol to further minimize radiation exposure, with a tube voltage of 100 kVp, and tube current modulation capped at 40 mA. Attenuation correction was performed using the manufacturer’s standard CT-based correction methods, ensuring precise alignment and quantification. This protocol was designed to minimize radiation exposure while providing high-quality imaging data to support dosimetry analysis.

### Image analysis

CT-based ROIs for normal organs were generated automatically using an in-house deep learning solution, which was developed and validated by the Core Facility for Automated Medical Image Contouring and Anatomical Analysis at our institution. This approach significantly reduces manual effort and variability associated with traditional contouring practices [34–36].

**Fig. 2:**
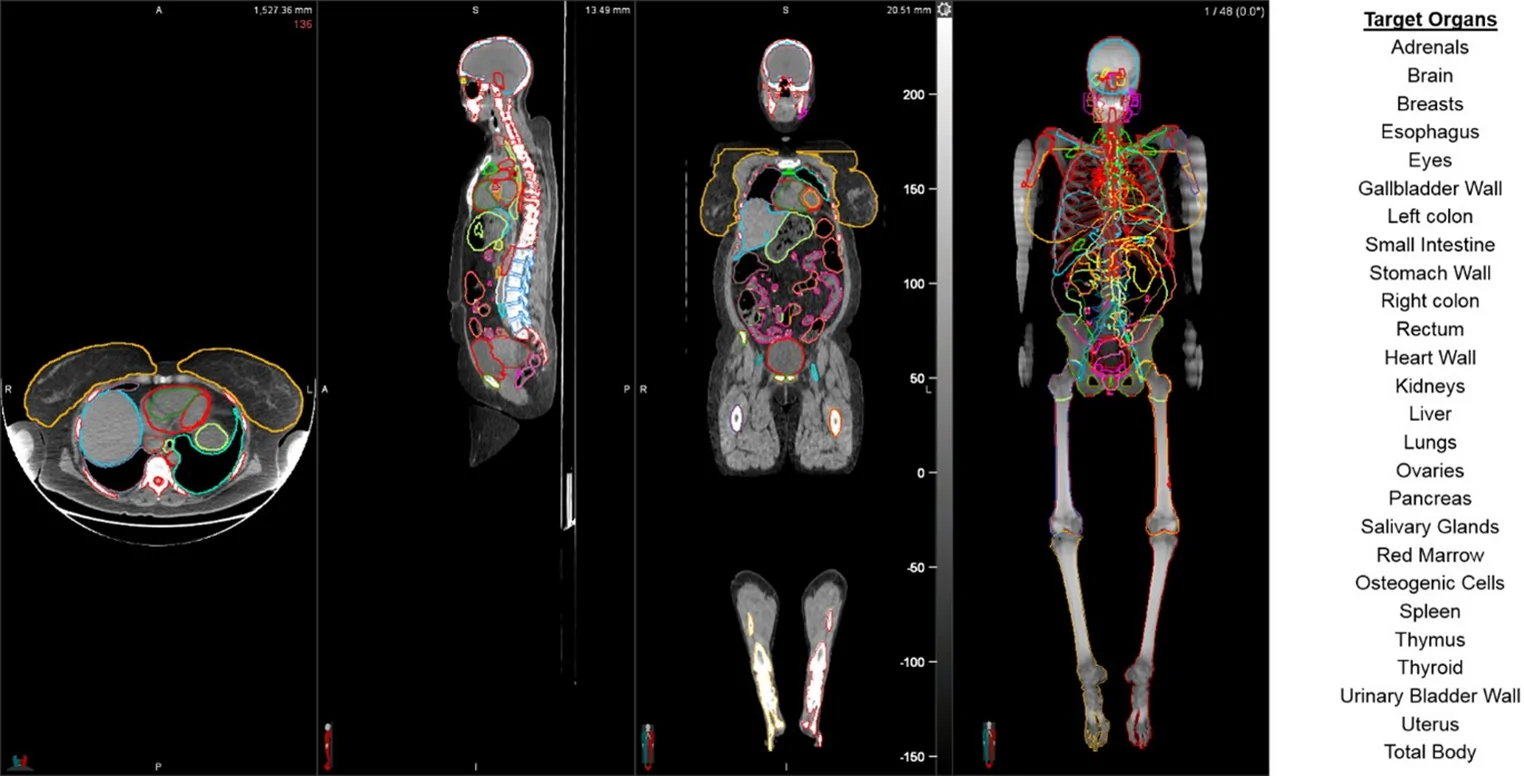
Semi-automated Organ Contouring for dosimetry analysis. Regions of interest were generated using an automated CT-based segmentation approach for dosimetry estimation. Axial, sagittal, coronal, and maximum intensity projection views are shown, with color-coded organ labels

Initial ROIs were generated using the non-contrast CT acquired with the initial PET/CT scan. A representative example is shown in Fig. 2. Digital Imaging and Communications in Medicine (DICOM) RT structure files were imported into MIM Encore 7.3 (Cleveland OH, USA) using a custom workflow for visualization and analysis. All automatically generated ROIs were visually reviewed and manually adjusted as needed on the initial CT scan and subsequently propagated to later imaging time points, with alignment refined using PET and low-dose CT images.

The time-activity data were extracted for each ROI, including total radioactivity (Bq), mean radioactivity concentration (Bq/mL), and volume (mL), across all imaging time points. These data were exported in .csv format for further analysis. Time-integrated activity coefficients (TIACs, MBq*h/MBq) were calculated using Simpson’s numerical integration method based on five time points. Beyond the final imaging time point (72 h), activity was extrapolated assuming physical decay only, consistent with EANM dosimetry guidance for early-phase studies, although this may overestimate residence time (Eq. 1). TIACs were imported into OLINDA 2.1 (Hermes Medical, Greenville, NC, USA) to compute organ-level dose coefficients (mGy/MBq) and effective-dose coefficients (mSv/MBq) based on standard adult female phantoms and ICRP 103 tissue weighting factors (Eq. 2) (32, 37–39).

Final ROIs and corresponding PET/CT datasets were archived in DICOM format to ensure data integrity and reproducibility.

1 $$\begin{aligned}\:\tilde{A}\left({r}_{s}\right)=\:{\int\:}_{0}^{\infty\:}A\left({r}_{s},t\right)dt\end{aligned}$$

where $\:A\left({r}_{S}\right)\:$denotes the activity in the source region as a function of time.

2 $$\:D\left({r}_{T},{T}_{D}\right)=\sum\:_{{r}_{s}}{\int\:}_{0}^{\infty\:}A\left({r}_{s},t\right)S\left({r}_{T}\leftarrow\:{r}_{s}\right)dt\:\:\:\:$$

where $\:D\left({r}_{T},{T}_{D}\right)$ is the absorbed dose to target region $\:T\:$where $\:\stackrel{\sim}{A}({r}_{S},\mathrm{t})\:$is the time-integrated activity in the source region $\:{r}_{S}$, and $\:S({r}_{T}\leftarrow\:{r}_{S})$is the S-value representing the mean absorbed dose to the target region per unit cumulated activity in the source region.

### Data analysis

Descriptive statistics are reported as mean ± standard deviation and median (range). A two-sided one-sample *t* test was used to compare the measured effective-dose coefficient of [^89^Zr]Zr-oxine-labeled leukocytes with a prespecified predicted adult female value (1.14 mSv/MBq) derived from package-insert dosimetry assumptions for [^111^In]In-oxine–labeled leukocytes [30]. Statistical analyses were performed using Python with SciPy. A two-sided *P* value < 0.05 was considered statistically significant.

## Results

### Participant characteristics and administered activity

Study participant demographics are summarized in Table 2. This study included four healthy adult females (mean ± standard deviation: 42.3 ± 7.4 years; range 37 to 53 years) who received [^89^Zr]Zr-oxine-labeled autologous leukocytes (mean ± standard deviation: 10.9 ± 2.9 MBq; range 9.3 to 15.2 MBq). All participants self-reported as White/Caucasian. Participant weight ranged from 54 to 86 kg (mean ± standard deviation: 68.8 ± 13.6 kg).

**Table 2:** Study participant demographics

Subject #	Sex	Age (Years)	Race/Ethnicity	Weight (kg)
1	Female	53	White/Caucasian	86
2	Female	41	White/Caucasian	63
3	Female	37	White/Caucasian	72
4	Female	38	White/Caucasian	54
Mean ± SD	-	42.3 ± 7.4	-	68.8 ± 13.6

Note. Race and ethnicity were self-reported by participants

### Blood analysis

**Fig. 3:**
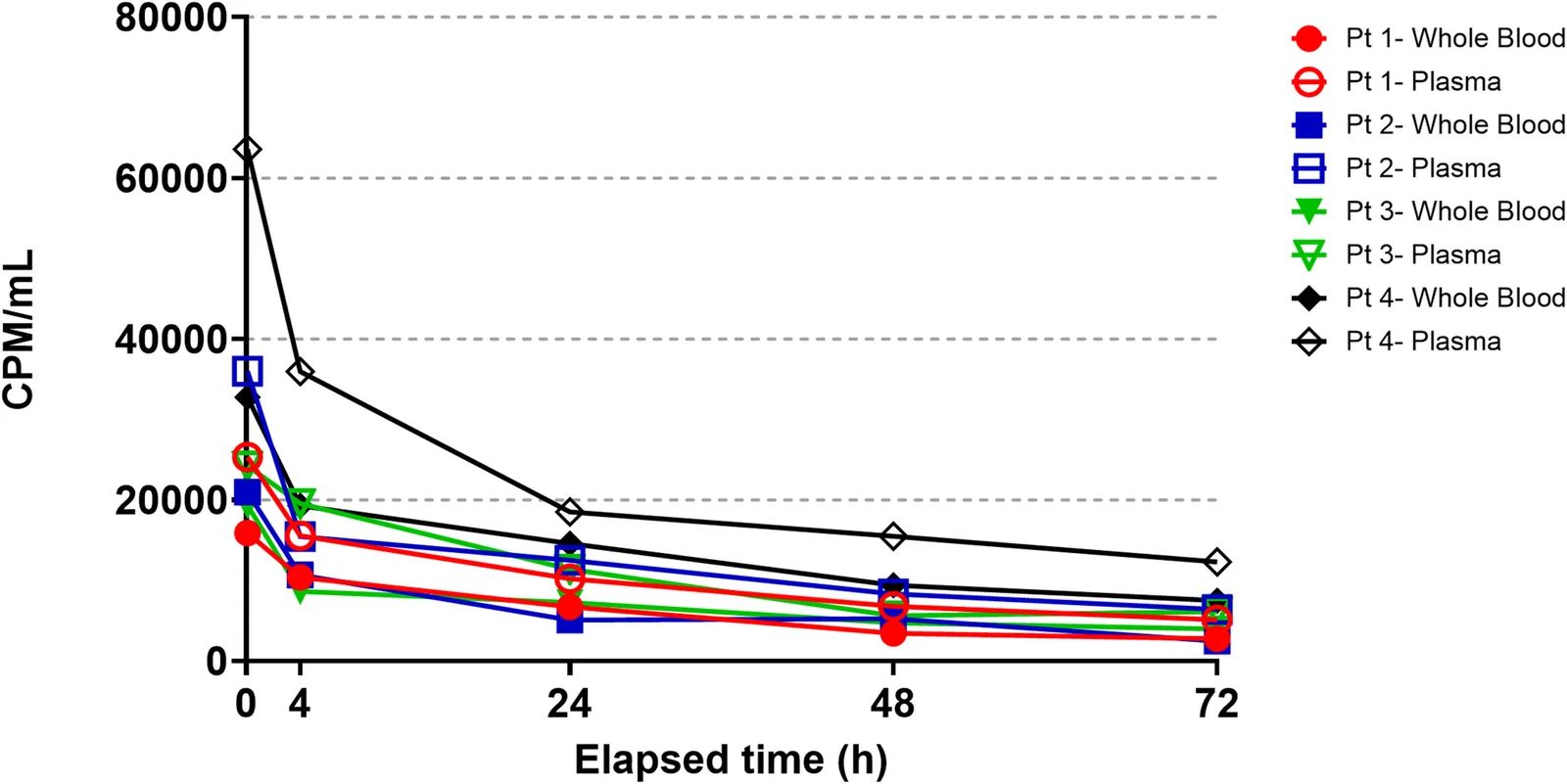
Measured activity (CPM/mL) in whole blood vs. plasma from each patient at each blood draw time

**Fig. 4:**
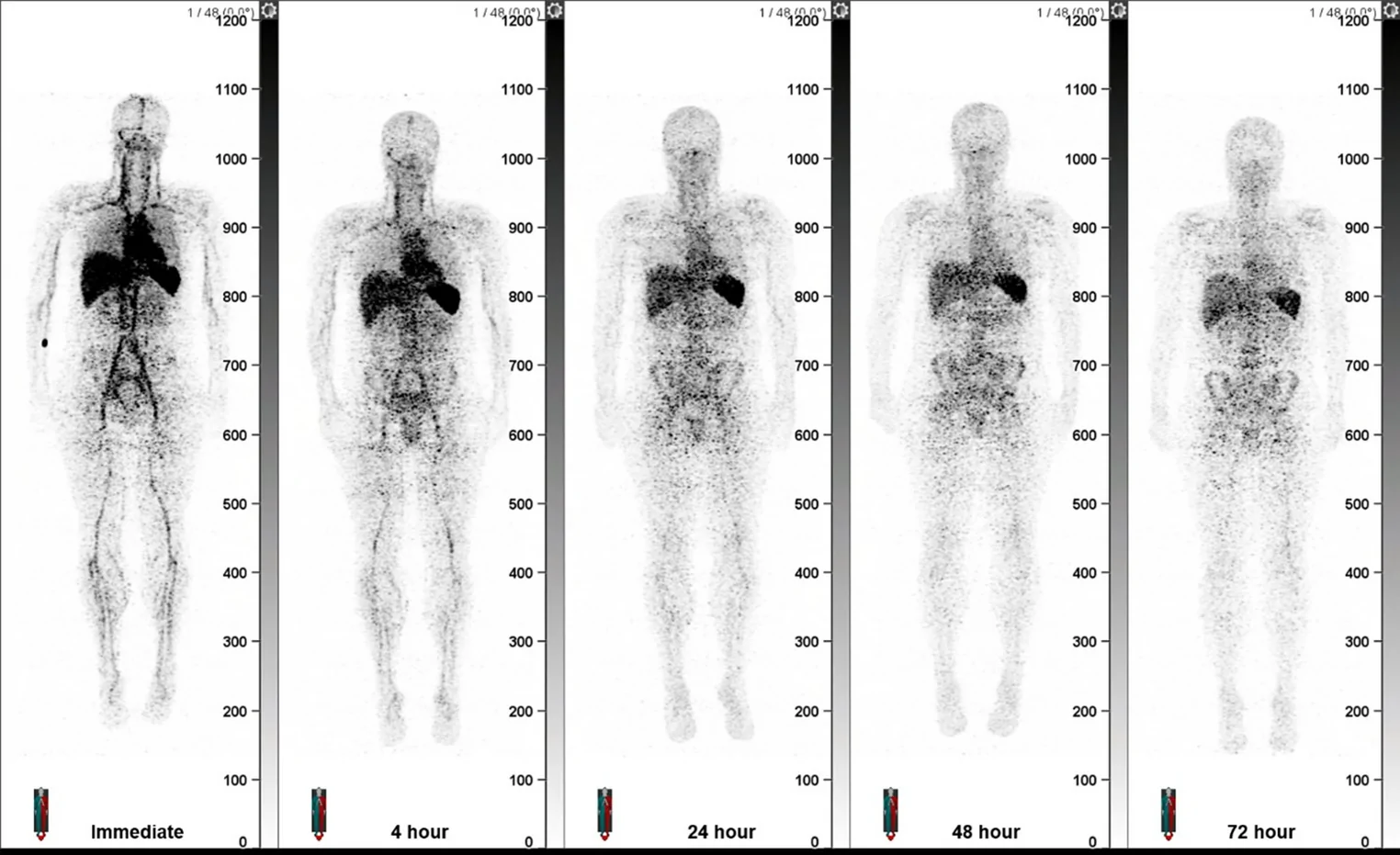
Representative Maximum Intensity Projection (MIP) PET images showing the biodistribution of [^89^Zr]Zr-oxine-labeled leukocytes at the five-imaging time points (0, 4, 24, 48, and 72 h) in a 53-year-old woman. The highest activities at later time points are observed in the spleen, bone marrow, and liver. Images are displayed on the same intensity scale in Bq/mL

Figure 3 shows the activity level (CPM/mL) in whole blood versus plasma in each patient at each time point. On average, activity levels in whole blood reduced from approximately 445 kBq/mL (4.05% of injected activity/mL) at the beginning to 83.56 kBq/mL (0.76% injected activity/mL) at 72 h, showing a decrease of about 81%. Similarly, there was an average reduction of about 80% in plasma activity levels, which dropped from 746 kBq/mL (6.78% of injected activity/mL) to 150 kBq/mL (1.36% injected activity/mL). Of note, the plasma concentration of activity was higher than the whole blood activity, especially at the initial time point. These findings are consistent with the PET images shown in Fig. 4, which show clearance of the labeled leukocytes from the blood pool over time.

### Predicted [^89^Zr]Zr-oxine autologous leukocytes` dosimetry

Predicted dosimetry for [^89^Zr]Zr-oxine autologous leukocytes was estimated assuming a biodistribution pattern based on published dosimetry for [^111^In]In-oxine leukocytes as described in the package insert. The assumed distribution included 30% to the spleen, 30% to the liver, 34% to red marrow, and 6% to the remainder of the body, with no excretion [30]. In this study, the measured organ biodistribution of [^89^Zr]Zr-oxine autologous leukocytes showed approximately 32% of activity in the liver, 8% in the spleen, and 15% in the red marrow. The relative contribution of individual organs to the total residence time of [^89^Zr]Zr-oxine autologous leukocytes is summarized in Figure S1. These residence time data were entered into OLINDA 2.1 to calculate organ dose-equivalent and effective-dose coefficients, considering only the physical decay of ^89^Zr beyond the final imaging time point without accounting for biological clearance, which may have resulted in a conservative overestimation of residence times and organ doses.

### Biodistribution and dosimetry

**Fig. 5:**
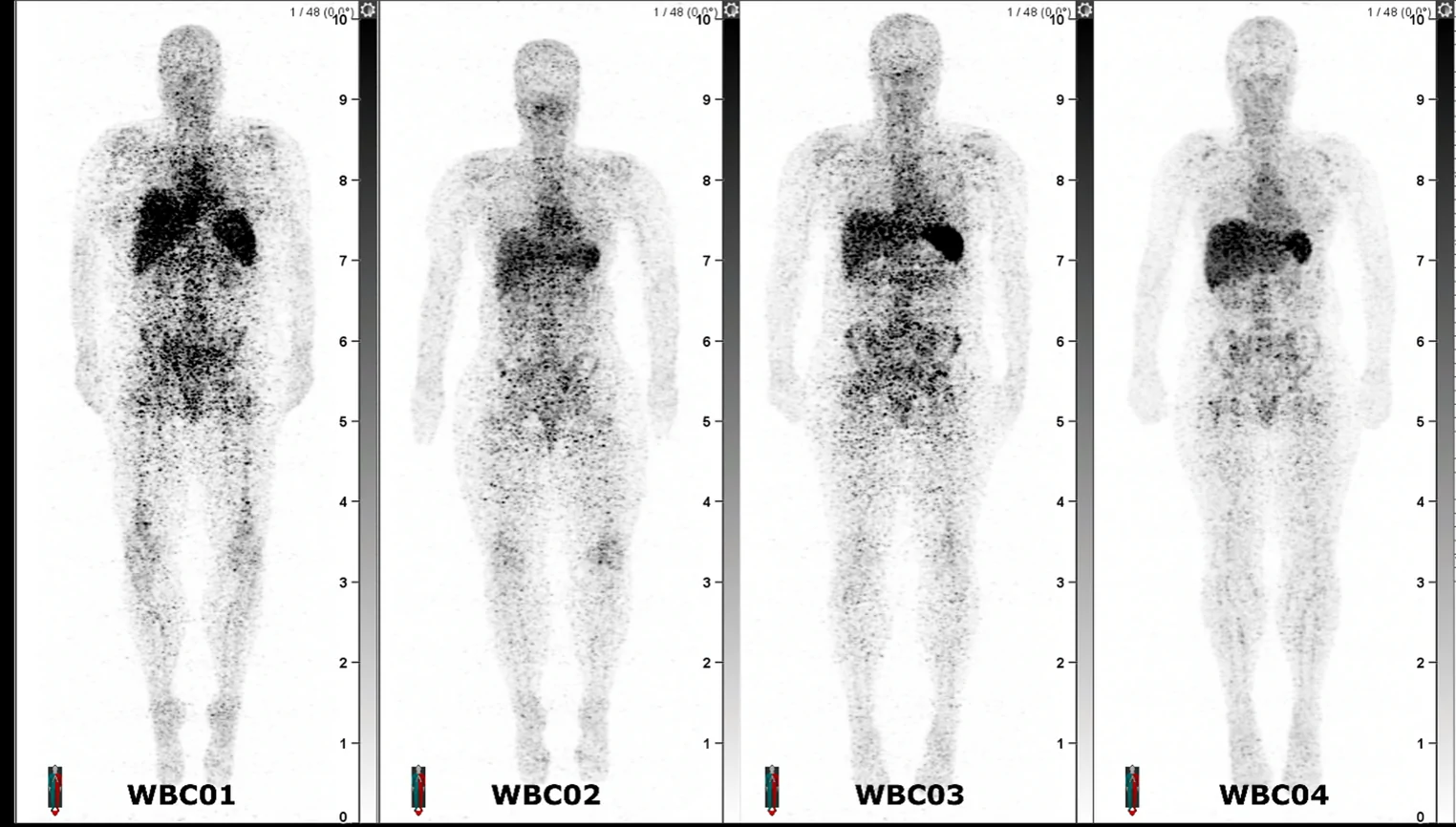
Comparative 48-hour maximum intensity projection PET images from all four healthy participants following administration of [^89^Zr]Zr-oxine-labeled autologous leukocytes. Images are displayed using an SUV scale of 0–10 to facilitate comparison across participants and demonstrate the reproducibility of biodistribution patterns, with predominant uptake in the liver, spleen, and bone marrow

After injection of [^89^Zr]Zr-oxine-labeled autologous leukocytes, participants were imaged with PET/CT at five imaging time points (0, 4, 24, 48, and 72 h). Representative whole-body PET images for one subject are shown in Fig. 4. At the initial imaging point (0 h) and continuing through 24 h, the highest uptake was observed in the liver, spleen, heart, and blood pool (Fig. 4). By 24 h and continuing through the 48- and 72-hour imaging time points, uptake in the liver, spleen, and red marrow, particularly within the pelvis and iliac crests, became increasingly prominent (Fig. 4). The overall biodistribution pattern was qualitatively similar to [^111^In]In-oxine-labeled leukocytes. Figure 5 demonstrates the reproducibility of these biodistribution patterns across all four healthy participants, with consistent predominant uptake in the liver, spleen, and bone marrow at 48 h.

To further evaluate the possibility of ^89^Zr efflux, an exploratory quantitative skeletal uptake analysis was performed (Supplementary Figure S2 and Supplementary Table S6). SUV_mean_ remained consistently higher within central marrow-rich skeletal regions than cortical tibial bone throughout the imaging period, while cortical tibial uptake remained low without evidence of progressive accumulation over time.

**Fig. 6:**
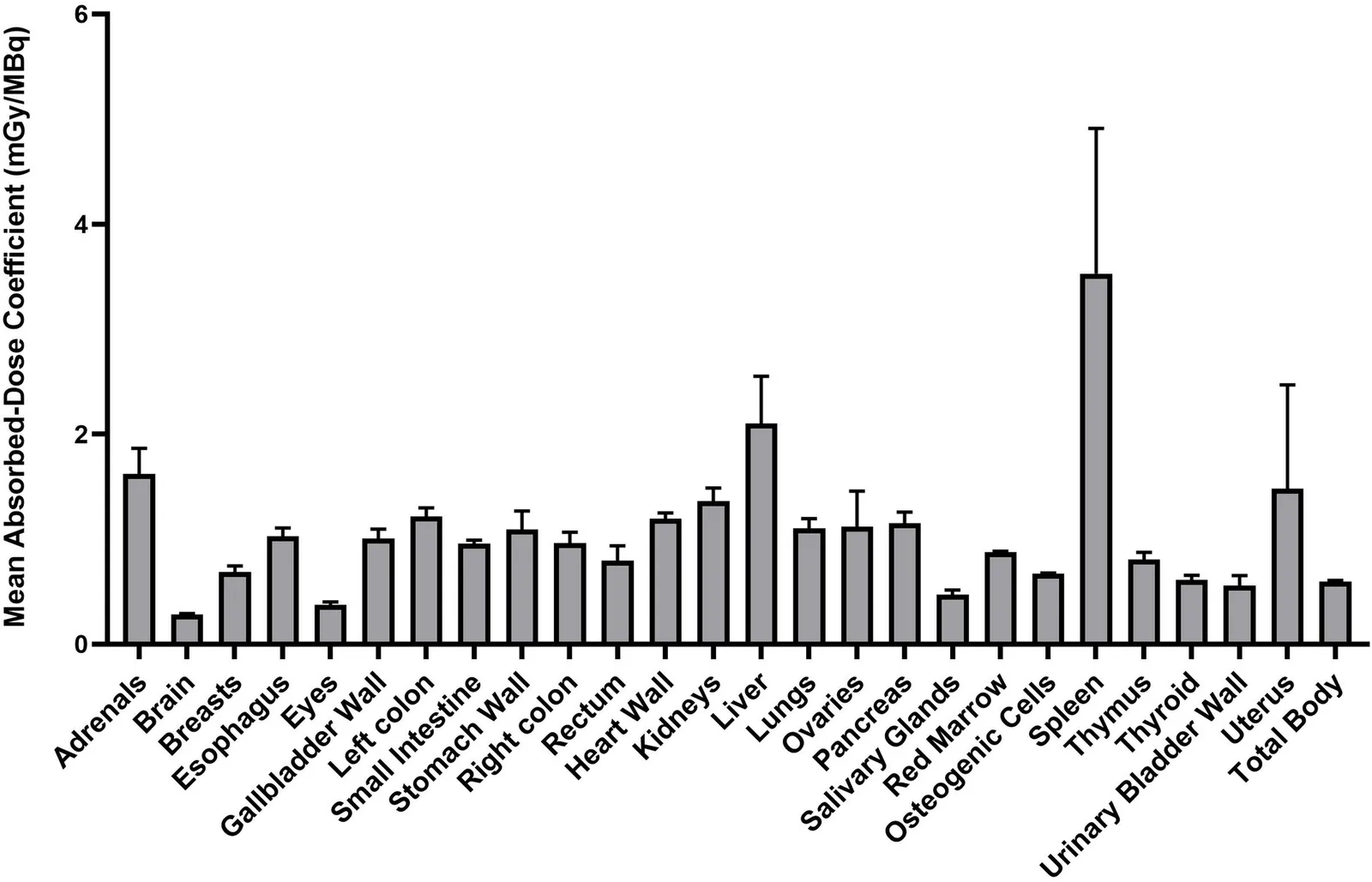
Mean absorbed-dose coefficient per organ. Bar graph shows mean absorbed-dose coefficients (mGy/MBq) with standard deviation for major organs in four participants. The highest absorbed-dose coefficients were observed in the spleen and liver

**Fig. 7:**
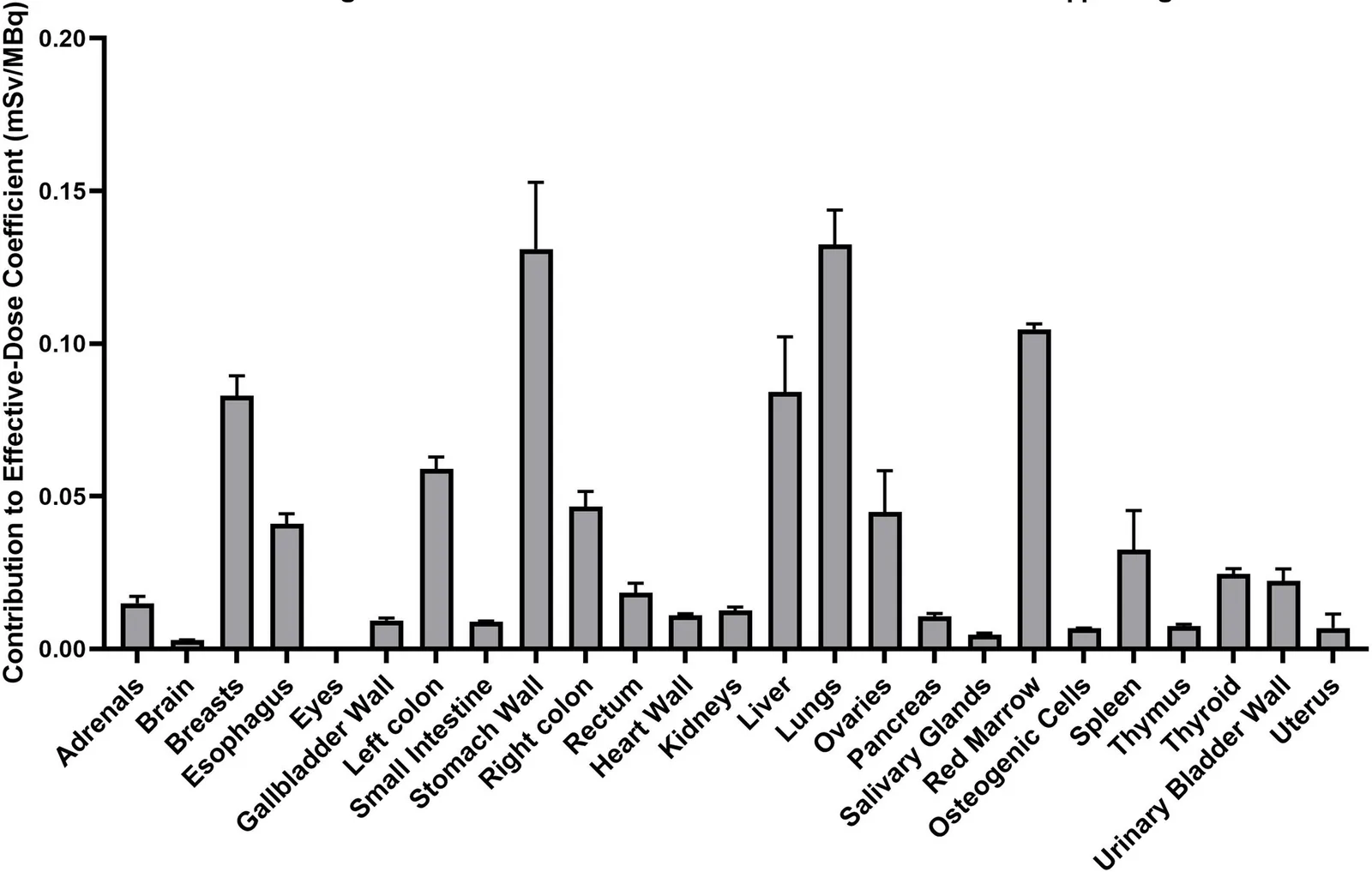
Contribution of each organ to the effective-dose coefficient. Bar graph shows the mean contribution of individual organs to the effective-dose coefficient (mSv/MBq) across all participants, with error bars indicating standard deviation. Organs with the largest contributions include the stomach wall, lungs, red marrow, and liver

The mean absorbed-dose coefficient per organ in mGy/MBq for the cohort is summarized in Fig. 6. The organs with the highest mean absorbed-dose coefficients included the spleen (3.52 ± 1.38), liver (2.1 ± 0.45), adrenals (1.62 ± 0.24), uterus (1.48 ± 0.98), and kidneys (1.36 ± 0.12). Additionally, cohort-level summary statistics for the contribution of each organ to the effective-dose coefficient are presented in Table 3. The average contribution of each organ to effective-dose coefficient in mSv/MBq for the cohort is summarized in Fig. 7. Organs with the highest contribution to effective-dose coefficient included the stomach wall (0.13 ± 0.02), lungs (0.13 ± 0.01), red marrow (0.10 ± 0.001), breasts (0.08 ± 0.006), and liver (0.08 ± 0.01) [37–39].

Individual organ absorbed-dose coefficients and average contribution to the effective-dose coefficient for each participant are provided in Supplementary Tables S2–S5.

**Table 3:** Cohort-level summary statistics of organ contributions to the effective-dose coefficient

All Cohort			
	Contribution to Effective Dose per organ (mSv/MBq)		
Target Organ	Median	Mean	SD
Adrenals	1.49E-02	1.50E-02	1.94E-03
Brain	2.80E-03	2.82E-03	1.00E-04
Breasts	8.26E-02	8.28E-02	5.75E-03
Esophagus	4.04E-02	4.10E-02	2.81E-03
Eyes	0.00E + 00	0.00E + 00	0.00E+00
Gallbladder Wall	9.13E-03	9.30E-03	7.00E-04
Left colon	5.81E-02	5.90E-02	3.36E-03
Small Intestine	8.89E-03	8.84E-03	2.56E-04
Stomach Wall	1.28E-01	1.31E-01	1.89E-02
Right colon	4.52E-02	4.67E-02	4.27E-03
Rectum	1.81E-02	1.84E-02	2.74E-03
Heart Wall	1.08E-02	1.10E-02	4.64E-04
Kidneys	1.23E-02	1.26E-02	1.01E-03
Liver	7.92E-02	8.42E-02	1.56E-02
Lungs	1.28E-01	1.33E-01	9.71E-03
Ovaries	3.90E-02	4.49E-02	1.17E-02
Pancreas	1.06E-02	1.07E-02	8.29E-04
Salivary Glands	4.74E-03	4.73E-03	3.81E-04
Red Marrow	1.05E-01	1.05E-01	1.48E-03
Osteogenic Cells	6.75E-03	6.71E-03	7.23E-05
Spleen	3.11E-02	3.25E-02	1.11E-02
Thymus	7.42E-03	7.45E-03	5.33E-04
Thyroid	2.40E-02	2.46E-02	1.46E-03
Urinary Bladder Wall	2.14E-02	2.23E-02	3.43E-03
Uterus	5.75E-03	6.84E-03	3.95E-03

The calculated mean effective-dose coefficient for female participants was 0.92 ± 0.06 mSv/MBq (median, 0.91 mSv/MBq; *n* = 4) with individual effective-dose coefficients for each participant presented in Table 4. Based on the previously published dosimetry for [^111^In]In-oxine-labeled leukocytes, the effective-dose coefficient for [^89^Zr]Zr-oxine leukocytes was predicted to be 0.855 mSv/MBq for adult males and 1.14 mSv/MBq for adult females. The measured effective-dose coefficient was numerically lower than the predicted value (mean difference, − 0.22 mSv/MBq; 95% CI, − 0.32 to − 0.12; t = − 6.98; *P* = 0.006). Given the limited sample size, this statistical comparison should be interpreted as exploratory.

**Table 4:** Effective dose coefficient, administered activity, and total effective dose for [^89^Zr]Zr-oxine labeled autologous leukocytes. Data are reported as the median and mean ± SD (namest = 4)

Subject #	Effective dose coefficient (mSv/MBq)	Administered Activity (MBq)	Effective dose (mSv)
1	1.00	9.25	9.25
2	0.86	9.25	7.97
3	0.87	10.00	8.78
4	0.94	15.17	14.26
**Median**	**0.9**	**9.63**	**9.02**
**Mean ± SD**	**0.92 ± 0.06**	**10.9 ± 2.9**	**10.07 ± 2.46**

### Safety assessment

The mean and standard deviation of the administered mass of 8-hydroxyquinoline was 1.4 ± 0.2 mg. The mean administered activity was 10.9 ± 2.9 MBq. No adverse events, clinically significant laboratory abnormalities, or detectable pharmacological effects were observed in any of the four participants following administration of [^89^Zr]Zr-oxine-labeled autologous leukocytes through 72 h post-administration. No clinically meaningful changes in vital signs were observed. Additionally, subjects were contacted 24 h after completing the protocol to assess any adverse events, and none were reported.

## Discussion

This first-in-human study establishes initial safety, biodistribution, and radiation dosimetry data of [^89^Zr]Zr-oxine-labeled autologous leukocytes, a PET imaging agent with potential applications in tracking immune cell dynamics in vivo. Importantly, this study represents a critical translational milestone by providing the first human safety, biodistribution, and dosimetry data necessary to support future clinical applications of PET-based leukocyte imaging in infection, inflammation, and cell-based therapies. Unlike PET agents with short-lived radionuclides (e.g. ^11^C, ^68^Ga, ^18^F) and/or rapid biological clearance, the longer half-life of ^89^Zr and retention of labeled leukocytes present potential dosimetry issues for imaging in people with long life expectancy, particularly for multiple studies in the same individual. These results support the safety of this approach and demonstrate a dosimetry profile within the range reported for other ^89^Zr-labeled radiopharmaceuticals. The observed biodistribution of [^89^Zr]Zr-oxine-labeled autologous leukocytes was consistent with prior murine [23, 24] and non-human primate [26, 27] preclinical studies, with predominant localization to the liver, spleen, and red marrow. This distribution pattern is also in agreement with established biodistribution of [^111^In]In-oxine-labeled leukocytes [30]. Unexpectedly, higher concentrations of activity were observed in the plasma compared to the whole blood which may have been due to leukocyte lysis, efflux of activity from leukocytes, or inadequate buffy coat formation with some leukocytes retained in the plasma. This phenomenon warrants further investigation in subsequent human studies.

The observed lower effective-dose coefficient compared with predicted values based on [^111^In]In-oxine–labeled leukocytes may reflect differences in both physical and biologic factors between the tracers. The PET/CT-based whole-organ dosimetry in this study differs from the SPECT only method used for In-111 dosimetry and is likely the major cause of the observed differences. Other potential sources of differences include differential intracellular retention of ^89^Zr-oxine over time by leukocytes compared with ^111^In-oxine.

Other promising techniques for cell labeling with [^89^Zr] exist including cell surface protein modification with deferoxamine (e.g., Df-Bz-NCS), and other chelator-based approaches, many of which show promising preclinical results [24, 25, 30, 40]. These methods label cell surface proteins rather than enabling intracellular trapping, which may result in differences in labeling stability, biodistribution, and in vivo cell tracking performance, and potentially cell function and behavior through modification of membrane-associated proteins. In contrast, [^89^Zr]Zr-oxine labeling allows intracellular delivery of the radionuclide, potentially improving retention within leukocytes and reducing label dissociation, although radionuclide efflux may still occur over prolonged imaging periods. Further comparative studies are needed to directly evaluate the relative advantages and limitations of intracellular versus surface-labeling strategies including their effects on cell viability, function, labeling stability, and quantitative imaging performance for clinical leukocyte imaging.

Free ^89^Zr released from labeled cells is known to exhibit high affinity for hydroxyapatite and therefore preferentially accumulates in mineralized bone [21, 40, 41]. Consequently, progressive uptake within cortical bone over time would be expected if substantial radionuclide efflux occurred in vivo. To further investigate this possibility, we performed an exploratory quantitative skeletal uptake analysis using the existing PET/CT dataset (Supplementary Figure S2 and Supplementary Table S6), comparing uptake within the central marrow-rich compartment of the spine and pelvis with cortical tibial bone. Uptake remained consistently higher within the marrow-rich compartment, whereas cortical tibial uptake remained low throughout the imaging period without evidence of progressive cortical accumulation. These findings support the interpretation that the observed skeletal activity predominantly reflects physiologic leukocyte homing to red marrow rather than redistribution of released free ^89^Zr. Nevertheless, low levels of radionuclide efflux cannot be completely excluded, particularly beyond the final imaging time point, and future studies incorporating longer imaging intervals or direct measurements of label stability would further characterize this phenomenon [15, 21, 40].

The [^89^Zr]Zr-oxine labeling approach was selected due to its similarity to the well-established ^111^In oxine labeling method, facilitating translational adaptation to clinical workflows. Recent first-in-human infection imaging using ^89^Zr-labeled leukocytes with a cell-surface chelator approach (Df-Bz-NCS) supports the clinical feasibility of PET-based leukocyte tracking and provides complementary clinical translation evidence to ex vivo ^89^Zr-labeling strategies [28]. This approach may also be well-suited for labeling cell-based therapies such as CAR T-cells to enable PET-based tracking of their initial biodistribution and tumor targeting during cancer therapy. Imaging cardiovascular inflammation (e.g. myocarditis, vasculitis) may be limited at early time points due to persistent activity in the blood pool and may require delayed imaging at 72 h or later for optimal target-to-blood pool contrast.

Comparing our findings with ^89^Zr-labeled monoclonal antibodies, we observe that the mean effective-dose coefficient of 0.92 ± 0.06 mSv/MBq for [^89^Zr]Zr-oxine-labeled autologous leukocytes is in the same range. For instance, the Phase I study of [^89^Zr]Zr-DFO-girentuximab, conducted on patients with clear cell renal cell carcinoma, reported an effective-dose coefficient of approximately 0.57 mSv/MBq [42]. Similarly, the first-in-human evaluation of [^89^Zr]Zr-pertuzumab in HER2-positive metastatic breast cancer patients demonstrated an effective-dose coefficient of 0.54 mSv/MBq, with the highest absorbed-dose coefficients observed in the liver, kidneys, and heart wall [43]. Although these agents differ biologically, this comparison provides a general reference for the magnitude of radiation exposure associated with ^89^Zr-based PET imaging. The higher dose we observed with [^89^Zr]Zr-oxine-labeled autologous leukocytes may be in part due to greater retention of activity in the body due to less metabolism and excretion of activity compared to monoclonal antibodies. The increasing availability of advanced long axial field-of-view PET/CT scanners from commercial vendors [44], which provide substantially higher sensitivity than conventional PET scanners, is expected to improve image quality and potentially reduce administered activity requirements, thereby further enhancing the utility of [^89^Zr]Zr-oxine-labeled autologous leukocytes.

An additional strength of this study is the use of an automated, CT-based organ segmentation workflow for dosimetry analysis. Automated segmentation approaches have been shown to reduce interobserver variability and improve reproducibility in quantitative imaging studies, which is particularly important for accurate estimation of organ-level radiation dose. This methodology may facilitate scalable and consistent dosimetry analyses in future studies involving larger cohorts or multi-institutional datasets [34–36].

The [^89^Zr]Zr-oxine PET approach may be particularly useful for tracking brain-infiltrating leukocytes in neuroinflammatory and neurodegenerative disorders. Brain-infiltrating leukocytes have been observed in animal models of stroke [45], traumatic brain injury [46], multiple sclerosis [47], and Alzheimer’s and Parkinson’s diseases [48]. In addition to BBB, these observations have not been replicated in living humans, due to the unavailability of proper imaging tools. Most PET-based radiopharmaceuticals have a half-life that is too short, as research suggests the critical window of initial leukocyte invasion is 3–4 days post-injury [49]. The better temporal and spatial resolution of [^89^Zr]Zr-oxine-labeled leukocytes may enable improved evaluation of the role of peripheral leukocyte infiltration into the central nervous system. Future studies will focus on applying this approach to patients with known or suspected CNS inflammatory conditions, including multiple sclerosis and Myalgic encephalomyelitis/chronic fatigue syndrome. Based on these initial promising results in healthy volunteers, further work to reduce the volume of blood required for consistent labeling and to increase the labeling efficiency with [^89^Zr]Zr-oxine is warranted.

This study has several limitations. First, the sample size was small, reflecting the early-phase, first-in-human design, which limits the statistical power and generalizability of the findings. Accordingly, the comparison between the measured and predicted effective-dose coefficients should be interpreted as exploratory. This sample size is typical of PET dosimetry studies and was intentionally small to limit the overall radiation exposure to healthy volunteers.

Second, only female participants were included. As females are predicted to have a higher effective-dose coefficient than males, the dosimetry could also be altered in patients with systemic infectious/inflammatory conditions compared to the healthy participants in this study.

In addition, time-integrated activity coefficients beyond the final 72-hour imaging time point were estimated assuming physical decay only. The study design excluding later time points was in part to reduce participant burden. This approach may overestimate residence times and absorbed doses for organs with continued biological clearance, particularly the liver and kidneys. However, this represents a conservative dosimetry assumption, as any continued biological clearance beyond 72 h would be expected to reduce, rather than increase, the estimated absorbed doses.

Although leukocyte viability was assessed during product release testing using trypan blue exclusion, additional functional characterization, including chemotaxis, phagocytic activity, activation status, and flow cytometry, was not performed. Because the labeling procedure was based on the established clinical methodology for FDA-approved [^111^In]In-oxine-labeled leukocytes, these studies were not required as part of the IND product release testing. Nevertheless, further evaluation of leukocyte functional integrity following ^89^Zr labeling would be valuable and should be investigated in future studies.

Finally, this study was performed in healthy volunteers and therefore does not capture potential alterations in leukocyte distribution that may occur in patients with active infection or inflammation. Future studies involving larger cohorts, both male and female participants, and patients with infectious or inflammatory diseases will be important to further validate these findings and assess their generalizability.

## Conclusion

This first-in-human dosimetry study with [^89^Zr]Zr-oxine-labeled autologous leukocytes was well tolerated. The observed biodistribution was consistent with previously conducted studies in murine and non-human primate models. The calculated effective-dose coefficient was consistent with the predicted effective-dose coefficient based on [^111^In]In-oxine in humans. These findings establish the first human safety, biodistribution, and dosimetry data for [^89^Zr]Zr-oxine-labeled autologous leukocytes and provide a foundation for future clinical investigations using PET-based immune cell tracking.

## Supplementary Information

Below is the link to the electronic supplementary material.

Supplementary Material 1 (105 KB DOC)

Supplementary Material 2 (119 KB DOCX)

## Data Availability

The datasets generated during and/or analyzed during the current study are available from the corresponding author upon reasonable request.
