# Supplementary material for: First-in-human dosimetry and biodistribution of [89Zr]Zr-oxine-labeled autologous leukocytes using PET/CT
Source: Eur J Nucl Med Mol Imaging. 2026 Oct 2;53(13):7786–98. doi: 10.1007/s00259-026-08158-w (PMC13727857; doi:10.1007/s00259-026-08158-w)
Supplement: Supplementary file 1 [file 259_2026_8158_MOESM2_ESM.docx]

**SUPPLEMENTAL DATA**

**First-in-Human Dosimetry and Biodistribution of [^89^Zr]Zr-oxine-Labeled Autologous Leukocytes Using PET/CT**

**Figure S1.** Percent contribution of individual organs to the total residence time of [^89^Zr]Zr-oxine autologous leukocytes. Organ-specific contributions were calculated from time-integrated activity coefficients obtained using serial whole-body PET/CT imaging and numerical integration. Percentages reflect mean values across the study cohort.

| Radiochemical purity | ≥ 95% |
| --- | --- |
| Labeling efficiency | 31.2% |
| Cell viability pre-label | 65.5 ± 9.3% |
| Cell viability post-label | 69.4 ± 5.4% |
| Cell viability 3 h | 74.6 ± 5.0% |
| Efflux (3 h) | 3.17 ± 2.53% |
| Sterility | Passed |
| Endotoxin | Passed |

**TABLE S1.** Radiolabeling quality-control results and product release specifications for [^89^Zr]Zr-oxine-labeled autologous leukocytes.

| **Subject 1**  **Target Organ** | **Beta** | **Gamma** | **Total**  **Absorbed Dose(mGy/MBq)** | **ICRP-103 Effective Dose(mSv/MBq)** |
| --- | --- | --- | --- | --- |
| Adrenals | 1.42E-01 | 1.74E+00 | 1.88E+00 | 1.74E-02 |
| Brain | 3.84E-02 | 2.38E-01 | 2.77E-01 | 2.77E-03 |
| Breasts | 2.38E-01 | 5.06E-01 | 7.44E-01 | 8.93E-02 |
| Esophagus | 2.43E-01 | 7.82E-01 | 1.02E+00 | 4.10E-02 |
| Eyes | 4.88E-02 | 2.83E-01 | 3.31E-01 | 0.00E+00 |
| Gallbladder Wall | 8.04E-02 | 8.45E-01 | 9.25E-01 | 8.54E-03 |
| Left colon | 3.39E-01 | 8.47E-01 | 1.19E+00 | 5.76E-02 |
| Small Intestine | 2.50E-01 | 7.15E-01 | 9.64E-01 | 8.90E-03 |
| Stomach Wall | 2.57E-01 | 1.07E+00 | 1.32E+00 | 1.59E-01 |
| Right colon | 1.88E-01 | 6.91E-01 | 8.79E-01 | 4.26E-02 |
| Rectum | 2.09E-01 | 7.62E-01 | 9.71E-01 | 2.23E-02 |
| Heart Wall | 2.59E-01 | 8.97E-01 | 1.16E+00 | 1.07E-02 |
| Kidneys | 2.79E-01 | 1.04E+00 | 1.32E+00 | 1.21E-02 |
| Liver | 6.41E-01 | 1.51E+00 | 2.15E+00 | 8.60E-02 |
| Lungs | 3.49E-01 | 7.34E-01 | 1.08E+00 | 1.30E-01 |
| Ovaries | 7.05E-01 | 9.19E-01 | 1.62E+00 | 6.49E-02 |
| Pancreas | 1.35E-01 | 1.04E+00 | 1.17E+00 | 1.08E-02 |
| Salivary Glands | 1.35E-01 | 3.63E-01 | 4.98E-01 | 4.98E-03 |
| Red Marrow | 2.78E-01 | 6.11E-01 | 8.89E-01 | 1.07E-01 |
| Osteogenic Cells | 1.51E-01 | 5.24E-01 | 6.75E-01 | 6.75E-03 |
| Spleen | 2.56E+00 | 2.57E+00 | 5.13E+00 | 4.73E-02 |
| Thymus | 1.75E-01 | 6.75E-01 | 8.50E-01 | 7.84E-03 |
| Thyroid | 1.42E-01 | 4.68E-01 | 6.09E-01 | 2.44E-02 |
| Urinary Bladder Wall | 1.65E-01 | 5.17E-01 | 6.81E-01 | 2.73E-02 |
| Uterus | 1.33E+00 | 1.47E+00 | 2.79E+00 | 1.29E-02 |
| Total Body | 1.20E-01 | 4.91E-01 | 6.11E-01 | 0.00E+00 |
| **Total Effective Dose** |  |  |  | 1.00E+00 |

**TABLE S2**. Dosimetry estimations for Subject One

| **Subject 2**  **Target Organ** | **Beta** | **Gamma** | **Total**  **Absorbed Dose(mGy/MBq)** | **ICRP-103 Effective Dose(mSv/MBq)** | |
| --- | --- | --- | --- | --- | --- |
| Adrenals | 1.28E-01 | 1.25E+00 | 1.37E+00 | 1.27E-02 |  |
| Brain | 3.74E-02 | 2.44E-01 | 2.82E-01 | 2.82E-03 |  |
| Breasts | 2.34E-01 | 4.98E-01 | 7.32E-01 | 8.78E-02 |  |
| Esophagus | 2.58E-01 | 7.35E-01 | 9.93E-01 | 3.97E-02 |  |
| Eyes | 8.70E-02 | 3.10E-01 | 3.97E-01 | 0.00E+00 |  |
| Gallbladder Wall | 1.50E-01 | 8.64E-01 | 1.01E+00 | 9.36E-03 |  |
| Left colon | 4.95E-01 | 8.34E-01 | 1.33E+00 | 6.44E-02 |  |
| Small Intestine | 2.48E-01 | 6.67E-01 | 9.15E-01 | 8.44E-03 |  |
| Stomach Wall | 1.47E-01 | 7.73E-01 | 9.20E-01 | 1.10E-01 |  |
| Right colon | 2.35E-01 | 7.18E-01 | 9.53E-01 | 4.62E-02 |  |
| Rectum | 1.68E-01 | 5.62E-01 | 7.29E-01 | 1.68E-02 |  |
| Heart Wall | 2.78E-01 | 8.89E-01 | 1.17E+00 | 1.08E-02 |  |
| Kidneys | 4.75E-01 | 1.06E+00 | 1.54E+00 | 1.42E-02 |  |
| Liver | 5.13E-01 | 1.30E+00 | 1.81E+00 | 7.23E-02 |  |
| Lungs | 3.43E-01 | 7.04E-01 | 1.05E+00 | 1.26E-01 |  |
| Ovaries | 2.89E-01 | 6.34E-01 | 9.23E-01 | 3.69E-02 |  |
| Pancreas | 1.35E-01 | 9.09E-01 | 1.04E+00 | 9.63E-03 |  |
| Salivary Glands | 1.39E-01 | 3.80E-01 | 5.20E-01 | 5.20E-03 |  |
| Red Marrow | 2.67E-01 | 5.90E-01 | 8.58E-01 | 1.03E-01 |  |
| Osteogenic Cells | 1.47E-01 | 5.28E-01 | 6.75E-01 | 6.75E-03 |  |
| Spleen | 1.09E+00 | 1.42E+00 | 2.51E+00 | 2.31E-02 |  |
| Thymus | 9.58E-02 | 6.47E-01 | 7.43E-01 | 6.86E-03 |  |
| Thyroid | 1.84E-01 | 4.92E-01 | 6.75E-01 | 2.70E-02 |  |
| Urinary Bladder Wall | 1.04E-01 | 3.72E-01 | 4.76E-01 | 1.90E-02 |  |
| Uterus | 8.61E-02 | 5.57E-01 | 6.43E-01 | 2.97E-03 |  |
| Total Body | 1.18E-01 | 4.75E-01 | 5.93E-01 | 0.00E+00 |  |
| Total Effective Dose |  |  |  | 8.62E-01 |  |

**TABLE S3**. Dosimetry estimations for Subject Two

| **Subject 3**  **Target Organ** | **Beta** | **Gamma** | **Total**  **Absorbed Dose(mGy/MBq)** | **ICRP-103 Effective Dose(mSv/MBq)** |
| --- | --- | --- | --- | --- |
| Adrenals | 2.44E-01 | 1.52E+00 | 1.77E+00 | 1.63E-02 |
| Brain | 3.54E-02 | 2.35E-01 | 2.71E-01 | 2.71E-03 |
| Breasts | 1.86E-01 | 4.53E-01 | 6.40E-01 | 7.68E-02 |
| Esophagus | 2.25E-01 | 7.22E-01 | 9.47E-01 | 3.79E-02 |
| Eyes | 9.19E-02 | 3.01E-01 | 3.93E-01 | 0.00E+00 |
| Gallbladder Wall | 1.41E-01 | 8.22E-01 | 9.63E-01 | 8.89E-03 |
| Left colon | 3.80E-01 | 8.26E-01 | 1.21E+00 | 5.85E-02 |
| Small Intestine | 2.89E-01 | 7.03E-01 | 9.92E-01 | 9.15E-03 |
| Stomach Wall | 1.41E-01 | 8.47E-01 | 9.87E-01 | 1.18E-01 |
| Right colon | 2.16E-01 | 6.94E-01 | 9.10E-01 | 4.41E-02 |
| Rectum | 1.82E-01 | 6.56E-01 | 8.38E-01 | 1.93E-02 |
| Heart Wall | 2.80E-01 | 8.83E-01 | 1.16E+00 | 1.07E-02 |
| Kidneys | 2.85E-01 | 9.60E-01 | 1.25E+00 | 1.15E-02 |
| Liver | 4.90E-01 | 1.24E+00 | 1.73E+00 | 6.94E-02 |
| Lungs | 3.48E-01 | 6.98E-01 | 1.05E+00 | 1.25E-01 |
| Ovaries | 3.13E-01 | 7.12E-01 | 1.02E+00 | 4.10E-02 |
| Pancreas | 1.65E-01 | 9.49E-01 | 1.11E+00 | 1.03E-02 |
| Salivary Glands | 9.99E-02 | 3.49E-01 | 4.49E-01 | 4.49E-03 |
| Red Marrow | 2.84E-01 | 5.94E-01 | 8.79E-01 | 1.05E-01 |
| Osteogenic Cells | 1.54E-01 | 5.20E-01 | 6.74E-01 | 6.74E-03 |
| Spleen | 2.08E+00 | 2.15E+00 | 4.23E+00 | 3.90E-02 |
| Thymus | 1.16E-01 | 6.41E-01 | 7.57E-01 | 6.99E-03 |
| Thyroid | 1.30E-01 | 4.60E-01 | 5.90E-01 | 2.36E-02 |
| Urinary Bladder Wall | 1.42E-01 | 4.47E-01 | 5.89E-01 | 2.36E-02 |
| Uterus | 6.88E-01 | 9.98E-01 | 1.69E+00 | 7.79E-03 |
| Total Body | 1.16E-01 | 4.71E-01 | 5.87E-01 | 0.00E+00 |
| **Total Effective Dose** |  |  |  | 8.78E-01 |

**TABLE S4.** Dosimetry estimations for Subject Three

| **Subject 4**  **Target Organ** | **Beta** | **Gamma** | **Total**  **Absorbed Dose(mGy/MBq)** | **ICRP-103 Effective Dose(mSv/MBq)** |
| --- | --- | --- | --- | --- |
| Adrenals | 1.04E-01 | 1.36E+00 | 1.47E+00 | 1.35E-02 |
| Brain | 4.81E-02 | 2.50E-01 | 2.98E-01 | 2.98E-03 |
| Breasts | 1.73E-01 | 4.72E-01 | 6.45E-01 | 7.74E-02 |
| Esophagus | 2.74E-01 | 8.62E-01 | 1.14E+00 | 4.55E-02 |
| Eyes | 8.24E-02 | 2.91E-01 | 3.74E-01 | 0.00E+00 |
| Gallbladder Wall | 1.24E-01 | 1.01E+00 | 1.13E+00 | 1.04E-02 |
| Left colon | 3.54E-01 | 7.85E-01 | 1.14E+00 | 5.53E-02 |
| Small Intestine | 2.66E-01 | 6.96E-01 | 9.62E-01 | 8.88E-03 |
| Stomach Wall | 2.27E-01 | 9.13E-01 | 1.14E+00 | 1.37E-01 |
| Right colon | 3.04E-01 | 8.03E-01 | 1.11E+00 | 5.37E-02 |
| Rectum | 1.26E-01 | 5.24E-01 | 6.51E-01 | 1.50E-02 |
| Heart Wall | 2.99E-01 | 9.80E-01 | 1.28E+00 | 1.18E-02 |
| Kidneys | 3.25E-01 | 1.02E+00 | 1.34E+00 | 1.24E-02 |
| Liver | 8.46E-01 | 1.87E+00 | 2.72E+00 | 1.09E-01 |
| Lungs | 4.37E-01 | 8.03E-01 | 1.24E+00 | 1.49E-01 |
| Ovaries | 2.96E-01 | 6.18E-01 | 9.14E-01 | 3.66E-02 |
| Pancreas | 1.91E-01 | 1.10E+00 | 1.29E+00 | 1.19E-02 |
| Salivary Glands | 8.68E-02 | 3.37E-01 | 4.24E-01 | 4.24E-03 |
| Red Marrow | 2.68E-01 | 6.01E-01 | 8.69E-01 | 1.04E-01 |
| Osteogenic Cells | 1.45E-01 | 5.13E-01 | 6.58E-01 | 6.58E-03 |
| Spleen | 9.37E-01 | 1.31E+00 | 2.24E+00 | 2.07E-02 |
| Thymus | 1.62E-01 | 7.16E-01 | 8.78E-01 | 8.10E-03 |
| Thyroid | 1.11E-01 | 4.72E-01 | 5.83E-01 | 2.33E-02 |
| Urinary Bladder Wall | 1.13E-01 | 3.66E-01 | 4.79E-01 | 1.92E-02 |
| Uterus | 1.96E-01 | 6.05E-01 | 8.01E-01 | 3.70E-03 |
| Total Body | 1.19E-01 | 4.84E-01 | 6.03E-01 | 0.00E+00 |
| **Total Effective Dose** |  |  |  | 9.40E-01 |

**TABLE S5.** Dosimetry estimations for Subject Four

These tables (S2-S5) present the absorbed dose (mGy/MBq) and effective dose (mSv/MBq) for subject one through four, according to ICRP-103 recommendations. Each table categorizes data by organ, detailing contributions from beta and gamma radiation, alongside their cumulative totals. The final column represents contributions to the effective dose from each organ, calculated using OLINDA software with tissue weighting factors from ICRP-103. The final row in each table indicates the total body effective dose accumulated.

**Figure S2. Exploratory quantitative skeletal uptake analysis following administration of [^89^Zr]Zr-oxine-labeled autologous leukocytes.**
**(A)** Mean SUVmean (±SD) measured within the central marrow-rich compartment of the spine and pelvis and within cortical tibial bone at each imaging time point.

**(B)** Mean central-to-peripheral SUVmean ratio over time.

The central marrow-rich compartment was generated by applying a 3-mm isotropic contraction to the original CT-based spine and pelvis bone segmentation. The peripheral mineralized-bone compartment was defined as the remaining outer 3-mm shell obtained by subtracting the contracted ROI from the original segmentation. Cortical tibial uptake was measured within a standardized 20-cm mid-diaphyseal (shaft) segment of the tibia. Across all imaging time points, activity remained highest within the marrow-rich compartment, whereas cortical tibial uptake remained low without evidence of progressive cortical accumulation.

| **Time** | **Central marrow-rich SUVmean** | **Peripheral mineralized-bone SUVmean** | **Central/peripheral SUVmean ratio** | **Cortical tibial SUVmean** | **Tibia/Central SUVmean ratio** |
| --- | --- | --- | --- | --- | --- |
| Immediate | 2.30 ± 0.06 | 1.94 ± 0.12 | 1.19 ± 0.07 | 0.35 ± 0.05 | 0.15 ± 0.02 |
| 4 h | 2.54 ± 0.32 | 2.02 ± 0.09 | 1.25 ± 0.11 | 0.44 ± 0.09 | 0.17 ± 0.03 |
| 24 h | 2.80 ± 0.47 | 2.16 ± 0.22 | 1.29 ± 0.10 | 0.43 ± 0.02 | 0.16 ± 0.03 |
| 48 h | 3.31 ± 0.32 | 2.45 ± 0.22 | 1.35 ± 0.05 | 0.51 ± 0.15 | 0.16 ± 0.05 |
| 72 h | 3.67 ± 0.41 | 2.65 ± 0.25 | 1.38 ± 0.06 | 0.43 ± 0.10 | 0.12 ± 0.03 |

**TABLE S6.** **Exploratory** **quantitative skeletal uptake analysis of [^89^Zr]Zr-oxine-labeled autologous leukocytes.** Mean ± SD SUVmean values measured within the central marrow-rich compartment, peripheral mineralized-bone compartment of the spine and pelvis, cortical tibial bone, and derived uptake ratios at each imaging time point.
